# Malaria Vaccines: Where Next?

**DOI:** 10.1371/journal.ppat.1000638

**Published:** 2009-10-30

**Authors:** Anthony A. Holder

**Affiliations:** Division of Parasitology, MRC National Institute for Medical Research, Mill Hill, London, United Kingdom; The Fox Chase Cancer Center, United States of America

A malaria vaccine for mass immunization, which could be delivered cheaply and widely and provide long lasting protection, would have a massive effect on global public health. However, such a vaccine does not currently exist. The reduction in global malaria incidence, in part due to the ongoing introduction of mosquito control measures such as insecticide-treated nets and disease treatment with artemesinin combination drug therapy, raises the performance requirements for a future vaccine, since the cost may be high relative to that of other interventions and the scale of the disease burden. Nevertheless, history teaches that complacency and an over-reliance on a small number of tools to combat this disease are dangerous. There is therefore still a pressing need for a vaccine to complement other control and potential elimination tools. The big question is whether or not a malaria vaccine that fulfills the above criteria can be developed.

RTS,S, the first malaria vaccine that targets the so-called pre-erythrocytic stages in humans, has just started in Phase 3 trials [1]; this is a major achievement based on numerous trials (most recent trials in [2],[3]) and 40 years of research (reviewed in [4]). Although questions persist about whether this vaccine will be effective enough [5] and about the nature of the protective mechanisms, does this achievement signal the end for research on new vaccines and the basic parasite biology and host immunology that underpins vaccine research? Other malaria vaccine candidates that target the asexual blood stages are not so advanced, and without improvement may not live up to hopes and expectations [6],[7]; is it therefore time to reassess the strategy on which they were developed [8]? For example, instead of subunit or vectored vaccines, the potential use of live or live and attenuated parasite vaccines has been proposed [9],[10] and is being pursued (http://www.sanaria.com/). Now that the international community is striving to eliminate malaria, maybe the requirements for a malaria vaccine are changing. Perhaps now is the time for a greater effort to research and develop the next generation of malaria vaccines. If this is the time for reinvigoration, what more do we need to know to develop malaria vaccines?

Current vaccines are based on a handful of proteins, several of which were first described several decades ago and before analysis of the *Plasmodium falciparum* genome indicated that there are about 5,400 protein-coding genes, some of which are expressed in an exquisitely stage-specific manner and others that are not. Are any of these other proteins antigens that are worth considering for vaccine development? It is unlikely that the level of resources devoted to the circumsporozoite surface protein (CSP) development that led to RTS,S could be mustered to support the further development of any new antigen. If all new malaria vaccines need to be compared against RTS,S, this could only be done in an expensive clinical trial format since alternative assays of efficacy do not exist. The cost and practicalities of this may inhibit vaccine-related research and the development of next generation vaccines, because a company or public–private partnership may not be prepared to put in the resources to produce a new vaccine. We must, therefore, ask where limited resources are best placed; vaccine discovery and development are both expensive. We have to take rational approaches; it's the best we can do, and resources are not available for purely empirical approaches. We need to enhance efforts on basic science in combination with clinical studies to provide a strong rationale for further vaccine development. This will need to be an integrated rather than a compartmentalized approach; vaccine development from antigen discovery to clinical trial is not a linear process. For example, clinical samples collected as part of a vaccine evaluation trial are essential for the development of better and more appropriate methods and assays to understand relevant human immune responses.

## Naturally Acquired Immunity—Do We Need to Do Better with a Vaccine?

The malaria parasite has co-evolved with its host over tens of thousands of years, so it is no surprise that it has a variety of mechanisms to evade the host immune response. *P. falciparum* also has a complex life cycle, and even now we are discovering new aspects of its biology. For a malaria vaccine, the big strategic questions remain: which stage or stages of the life cycle do we want to target, what are the targets, and how do we deliver them? Can a malaria vaccine mature the immune system from that of a naïve individual to that of an individual protected from death and disease? Can we do better than what is achieved in naturally acquired immunity? An increased focus on elimination of malaria has led to the suggestion that a transmission-blocking vaccine, either targeting sexual stages alone or sexual- and pre-erythrocytic stages, is all we will need in the future; this is optimistic because of the apparent lack of naturally acquired immunity to these stages and the potential lack of benefit to the individual vaccinee.

With time and exposure, individuals that survive a malaria infection develop immunity to the disease. The acquisition of immunity that protects against death and severe disease precedes that which protects against mild malaria [11]. Sterile immunity may never be achieved, which may not have consequences for the individual, but it is important for preventing transmission and eventually eliminating the parasite. Different strategies targeting different stages of the life cycle and the use of attenuated parasites or mixtures of several or single antigens in a variety of live, attenuated vector- or adjuvant-based delivery systems are all being investigated. This is the basis upon which the development of a malaria vaccine rests. Since in holoendemic areas it is young children rather than adults who are particularly at risk, we need a better understanding of the maturation of the immune system with age. This, together with immunological memory, is one area for immunologists to make significant contributions to the malaria field [12].

## Different Facets of Immunity

Effective protection involves both cellular and humoral immunity, with antibody being important in targeting free parasites and the blood stages, and a cellular response probably most important against pre-erythrocytic stages. However, the targets and the mechanisms still need further elucidation. Studies of immunity induced by experimental infection of humans will provide considerable insight into mechanisms of immunity and their targets [13]. The antigens are many and varied and most remain to be characterised. Some proteins undergo antigenic variation, others are highly polymorphic, and some are conserved in sequence. Antigenic variation has been described largely in the context of proteins on the infected red cell surface [14], but is also a feature of merozoite proteins involved in invasion and coded by small gene families [15]. Extensive sequence polymorphism is a feature of merozoite proteins such as merozoite surface protein 2 (MSP2) [16] and apical membrane antigen (AMA)1 [17], whereas parts of MSP1 seem to be highly conserved and yet also the target of protective antibodies [18]. We still don't know for certain whether immunity requires accumulation of responses to different forms of variant or diverse antigens, additive responses to a number of antigens [19], or the right response against a single antigen (for example, in terms of fine specificity, avidity, concentration, and class of antibody) [18]. If antigenic diversity is the primary driver of immune evasion, it may never be possible to develop a sufficiently complex vaccine. Whatever the delivery system used, the repertoire of antigens will be limited, so perhaps we need to focus on first establishing the feasibility of vaccination against a heterogeneous population of parasites. Using massively parallel sequencing technologies to survey the genome and transcriptome of wild parasites, and methods to measure the specificity and level of immune responses to many individual antigens [20] in single individuals of well-defined clinical status, together with informatics tools to analyse the data [21], will provide insights into this issue.

## A Focus on Merozoites

It has been proposed that acquisition of immunity to strain-transcending epitopes is a feature of natural infection [11]. If antigenic variation is therefore not of major significance in acquisition of immunity, then proteins on the surface of the infected red cell such as EMP1, rifins, and stevors may have little importance as vaccine candidates for the asexual blood stages. The focus is placed on the merozoite, in particular on proteins that are exposed to antibodies such as those on the surface, in the micronemes/rhoptries, and in the parasitophorous vacuole, including shed and soluble proteins. The duration of a protein's accessibility to antibody is an important criterion and may explain why the parasite sheds all or most of its surface proteins at invasion. Removal of immune complexes from the parasite surface will reduce susceptibility to Fc-mediated effector functions.

Most vaccines currently in or approaching clinical testing are based on antigens that were first described almost 30 years ago. It was first shown in 1981 that immunisation with a single protein (MSP1) protected against challenge infection in a rodent malaria model [22]. The genome/transcriptome/proteome projects of the last 10 years have identified the spectrum of proteins at different stages of the life cycle in which there are likely to be many potential new candidates that need to be evaluated [23]. Criteria or guidelines to assist in the prioritisation of antigens have been developed (see, for example, http://www.emvi.org/Portfolio/EC+Funded+Projects/EURHAVAC). Such guidelines can be obvious (but not always followed), for example, if it is proposed that a particular antigen is the target of antibodies it must be accessible at the appropriate time in the life cycle. We must accelerate the movement of ideas from basic science to the first step of potential development: funding for this has often been limited because it falls into the gap between basic and applied science.

Clearly, any strategy for vaccine development will need to focus equally on the delivery system to induce the right sort and level of response and the antigens to ensure that the response is effective. There is growing interest in immunisation by using live and attenuated parasites [24]–[26]. Provided that the practical issues of delivery and regulatory and ethical issues of safety can be overcome, then genetic modification of the parasite could provide immense benefit; for example, developing an in vitro system for sporozoite replication, developing a parasite expressing critical antigens normally expressed at other stages such as expressing the sexual stage antigen Pfs25 on the sporozoite surface, or using genetic inactivation techniques [27]. Parasites that have lost the ability to regulate *var* gene expression [28] or blood stage parasites attenuated by metabolic mutants [26] are potential vaccine candidates. Even if using parasites is a feasible approach, it still needs to be established that a single vaccine strain has the potential to deliver the diversity of sequence necessary if antigen diversity/variation is important.

## The Need for Good Assays

There are too few resources to follow an entirely empirical approach to vaccine discovery and development. Therefore, there needs to be a focus on the development of assays that may be predictive of protection in humans. In the absence of a vaccine that works through a known mechanism, there is no gold standard to establish and calibrate in vitro (or in vivo) assays that are predictive of a protective immune response. A conundrum is, how is it possible to do comparative studies of different vaccines with assays that are not validated? Recent developments in transgenic host and transgenic parasite technology [29],[30] represent an important achievement that may help out here, but may also yield some surprises. For example, studies using *Plasmodium berghei* expressing *P. falciparum* CSP suggest that CSP is not important in immunity provided by immunisation with attenuated sporozoites [31]. This is a surprising finding since the leading malaria vaccine candidate, RTS,S, is comprised of part of CSP in a virus-like particle and a strong adjuvant. In other studies, a role for antibody Fc-dependent mechanisms has been highlighted using passive immunization with human antibody of mice that are transgenic for human Fc receptors and challenged with a *P. berghei* that is transgenic for part of *P. falciparum* MSP1 [32]. However, as with other assays, the relevance to humans still needs to be established.

The priorities are to ascertain the basis of protection against malaria and develop delivery systems to elicit the right immunological response and memory. These delivery systems may be attenuated parasites, vectored vaccines, or adjuvant and subunit protein-based. No doubt new and better antigens will be identified and prioritised, and combinations will need to be tested, with different targets and effector mechanisms at different stages of the life cycle. All of this will need to be underpinned by basic biological studies.
